# Causal association between self-reported fatigue and coronary artery disease: a bidirectional two-sample Mendelian randomization analysis

**DOI:** 10.3389/fpsyt.2023.1166689

**Published:** 2023-09-20

**Authors:** Xiaoyi Qi, Shijia Wang, Liangxian Qiu, Xiongbiao Chen, Qianwen Huang, Kunfu Ouyang, Yanjun Chen

**Affiliations:** ^1^Departments of Cardiology, Peking University Shenzhen Hospital, Shenzhen, China; ^2^Medical College, Shantou University, Shantou, China; ^3^Department of Cardiovascular Surgery, Peking University Shenzhen Hospital, Shenzhen, China

**Keywords:** self-reported fatigue, psychiatry symptom, coronary artery disease, Mendelian randomization analysis, GWAS

## Abstract

**Background:**

Observational studies have reported the association between fatigue and coronary artery disease (CAD), but the causal association between fatigue and CAD is unclear.

**Method:**

We conducted a bidirectional Mendelian randomization (MR) study using publicly available genome-wide association studies (GWAS) data. The inverse-variance weighted (IVW) method was used as the primary analysis. We performed three complementary methods, including weighted median, MR-Egger regression, and MR pleiotropy residual sum and outlier (MR-PRESSO) to evaluate the sensitivity and horizontal pleiotropy of the results.

**Result:**

Self-reported fatigue had a causal effect on coronary artery atherosclerosis (CAA) (OR 1.047, 95%CI 1.033–1.062), myocardial infarction (MI) (OR 1.027 95%CI 1.014–1.039) and coronary heart disease (CHD) (OR 1.037, 95%CI 1.021–1.053). We did not find a significant reverse causality between self-reported fatigue and CAD. Given the heterogeneity revealed by MR-Egger regression, we employed the IVW random effect model. For the examination of fatigue on CHD and the reverse analysis of CAA, and MI on fatigue, the MR-PRESSO test found horizontal pleiotropy. No significant outliers were found.

**Conclusion:**

The MR analysis reveals a causal relationship between self-reported fatigue and CAD. The results should be interpreted with caution due to horizontal pleiotropy.

## 1. Introduction

Coronary artery disease (CAD) is a cardiovascular disorder that has become the leading cause of death in developed and developing countries (1). According to the Global Burden of Illness (GBD) study, cardiovascular disease was the primary factor in around one-third of all deaths worldwide (2). As an atherosclerotic disease, CAD includes coronary artery atherosclerosis (CAA), myocardial infarction (MI), and coronary heart disease (CHD) (3). In China, more than 1 million people die of CHD every year. By 2030, the World Bank predicts that there will be 23 million MI patients worldwide (4). With high morbidity and mortality, the identification of novel risk factors of CAD for early prevention and intervention is crucial (5). In epidemiological research, the importance of behavioral and psychosocial factors in the prevention, development, and treatment of CAD is gradually appearing.

In a GWAS study, self-reported fatigue was defined as tiredness and low energy during the last 2 weeks (6). Previous research has shown a bidirectional relationship between fatigue and coronary artery disease. On the one hand, fatigue is a typical symptom for patients with chronic diseases like CAD. Greater post-CVD fatigue was associated with increased mortality (HR 1.13, 95%CI 1.06–1.22) (7). Furthermore, in individuals with CAD, fatigue is a common disturbing symptoms, which affect cognitive performance and quality of life. A recent cross-sectional study indicated that higher subjective fatigue is significantly associated with less perceived social support in patients with established CAD (8, 9). On the other hand, self-reported fatigue is associated with blunted cardiovascular reactivity, which could further identify participants with an increased risk of adverse cardiovascular outcomes (10). The result from an early study revealed that participants with higher levels of fatigue reported weaker blood pressure, and heart rate responses to stress (11). A cross-sectional study also indicated that higher symptoms of persistent fatigue were related to attenuated blood pressure reactivity to acute stress (12). Besides, fatigue can be examined as a symptom or as part of psychological constructs of depression and vital exhaustion (13). A meta-analysis concluded that somatic depressive symptoms, including insomnia, fatigue, and work difficulty, were significantly associated with cardiovascular prognosis (HR 1.19, 95%CI 1.10–1.29) (14). Clinical evidence suggested that vital exhaustion was associated with an increased risk of adverse cardiovascular events (15). These results showed an indirect association between fatigue and cardiovascular events. However, there could exist bias, confounders, and reverse causality in observational studies. Besides, the causal relationship between fatigue and CAD is still unclear.

Mendelian randomization (MR) is a new approach to investigating the causal link between exposures and outcomes via genetic variants. Because genetic variants are typically irrelevant to confounding factors, differences in outcomes could be attributed to the difference in risk factors (16). To minimize reverse causality bias, we conducted a bi-directional MR investigation in this study to investigate the causal association between self-reported fatigue and CAD.

## 2. Method

### 2.1. Study design

**Figure 1:**
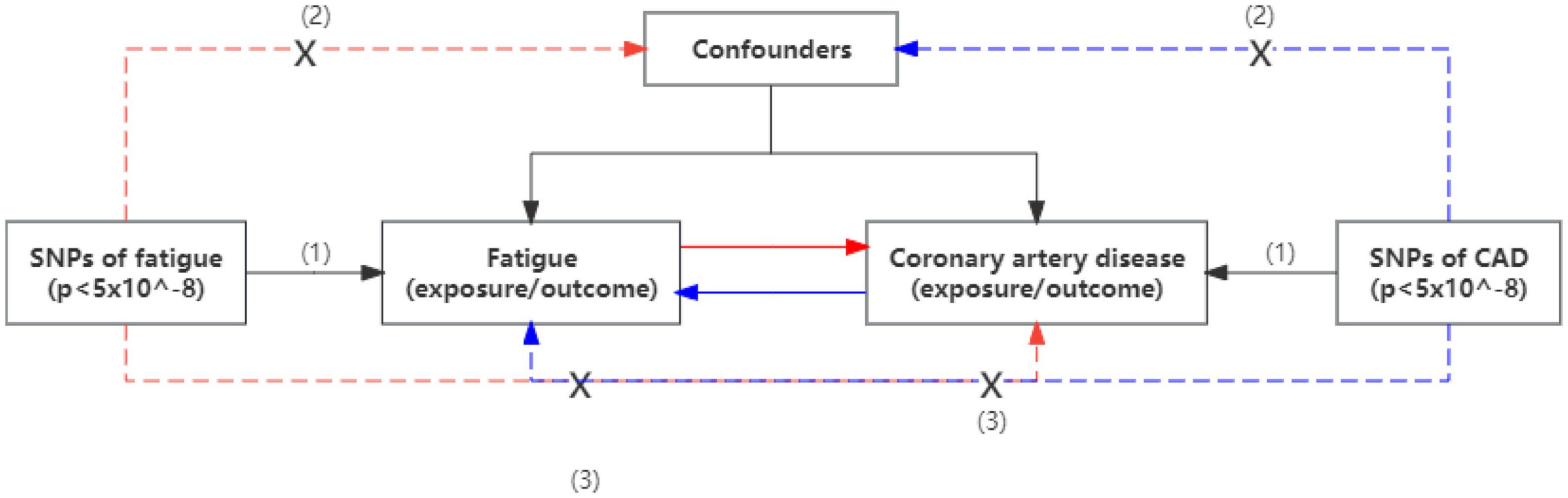
An overview of the study design. Assumption (1), instrumental variables directly affected exposure; Assumption (2), instrumental variables were not associated with other confounders; and Assumption (3), instrumental variables affect the risks of outcomes only through exposure, not through other pathways. SNP, single-nucleotide polymorphism; CAD, coronary artery disease.

We conducted a two-sample bidirectional Mendelian randomization study to assess the causal relationship between fatigue and coronary artery disease (Figure 1). The traits of coronary artery disease include coronary artery atherosclerosis (CAA), myocardial infarction (MI), and coronary heart disease (CHD). Summary-level GWAS statistic for fatigue and CAD was obtained from public GWAS studies. No additional ethics statement or consent was required. The following three conditions must be satisfied for our MR analysis to be valid: (1) the instrumental variables (IVs) are strongly associated with the exposure; (2) the IVs are not associated with any confounding factors which affect both exposure or outcome; and (3) the IVs affect the outcome only through exposure.

### 2.2. Data source and SNP selection

Genetic variants on fatigue were obtained from a GWAS study (Deart V. 2017) based on a UK Biobank sample. There were 449,019 participants in total. The study aims to investigate the genetic contribution to self-reported tiredness/fatigue. Participants would be asked the question: “Over the past 2 weeks, how often have you felt tired or have little energy?,” which was part of the Mental Health Questionnaire consisting of items from the Patient Health Questionnaire. To evaluate the frequency of fatigue/tiredness, four-variable responses like “Not at all/Several days/More than half the day/Nearly every day” would be utilized (6). The summary statistics of CAA were extracted from a GWAS in the UK Biobank, including 14,334 cases and 346,860 controls from European ancestry participants. As for the genetic variants of MI and CHD, we obtain both from different GWAS studies in the UK Biobank, including 7,018 cases and 354,176 controls, 10,157 cases, and 351,037 controls separately. Detailed information for the GWAS data source was shown in Table 1.

**Table 1:** Source of GWAS data.

Phenotype	GWAS data source	Sample size	Study population	Study ID
Fatigue	UK Biobank	449,019	European	ukb-b-929
Coronary artery atherosclerosis	UK Biobank	361,194	European	ukb-d-I9_CORATHER
Myocardial infarction	UK Biobank	361,194	European	ukb-d-I9_MI
Coronary heart disease	UK Biobank	361,194	European	ukb-d-I9_CHD

We conducted the following measures to ensure the selected IVs satisfy the three core MR assumptions. Firstly, we used genetically important *p*-value (*p* < 5×10^−8^) to select SNPs that were strongly associated with exposures. Secondly, the clumping procedure with *R*^2^ < 0.01 and a window size of 10,000 kb adherence to the European ancestry individuals were performed to exclude SNPs with linkage disequilibrium (LD). Thirdly, we would use proxy SNPs with high LD (*R*^2^ > 0.8) to substitute the missing SNPs in outcome GWAS. Fourthly, we harmonized the exposure and outcome dataset to eliminate SNPs with intermediate allele frequencies. Besides, to avoid weak instrument bias, we calculated the *F* statistics for each SNP with the equation: *F* = *R*^2^ (N−K−1) / [K (1−*R*^2^)]. IVs with *F*-statistics less than ten were considered weak instruments and would be excluded from further analysis (17).

### 2.3. Statistical analysis

Mendelian randomization (MR) is an analysis that investigates the causality between exposures and outcomes using genetic variants (16). In this MR study, we adopted the inverse-variance weighted (IVW) method as the primary analysis. The IVW method hypothesized that all genetic variants are valid and are most statistically robust when the average pleiotropic effect is zero (18). To further validate the stability of the results, several additional methods were performed to assess causal associations. The MR-Egger regression could estimate the causal effects from a weighted linear regression even in the presence of horizontal pleiotropy (19). The weighted median method was also performed, which could provide an unbiased estimate effect when at least half of the genetic variants are valid instruments (20).

Then a series of sensitivity analyses were performed. Because of the different sources of data, MR analysis may have heterogeneity. The heterogeneity was assessed by Cochran Q statistic and *p*-value. *p*-value < 0.05 was defined as significant heterogeneity. Horizontal pleiotropy means that genetic variation for exposure can affect outcomes through multiple independent pathways. The pleiotropic effects of IVs could be indicated by the intercept of MR-Egger regression (19). Additionally, the MR-PRESSO test was conducted to assess pleiotropy. The MR-PRESSO method could identify and correct outliers in the IVW linear regression. If the *p*-value of the global and distortion test indicated potential pleiotropy (*p* < 0.05), the causal estimates that have been corrected for outliers would be provided (21). Finally, the leave-one-out analysis was also applied to identify whether the effects of causal association would be affected by the removal of any single SNP (20).

The effect estimate for the causal associations between fatigue and CVD would be presented as odds ratios (ORs) with a 95% confidential interval (CI). The “TwoSampleMR” and “MR-PRESSO” packages of the R software, version 4.2.2, were used to perform this MR analysis.

## 3. Result

### 3.1. Characteristics of selected SNPs

For the causal effects of fatigue on CAD, we selected 35 fatigue-associated SNPs with a significant *p*-value of 5×10^−8^. The *F*-statistic value was all more than 10, with an average value of 13.24. The selected SNPs were all available in the outcome data. No proxy SNP was used. 1 SNP (rs2426132) was left out in the harmonization process because it is palindromic and has intermediate allele frequencies. Eventually, 34 SNPs were included for further MR analysis. Detailed information for selected SNPs were listed in Supplementary Table S1. For the reverse analysis, 34 CAA-associated SNPs were selected. The *F*-statistics for all SNPs were greater than 10, with an average value of 47.06. 1 CAA-associated SNPs were not available in the summary data of fatigue. In the harmonization process, 1 SNP (rs1010322) was removed for being palindromic and having intermediate allele frequencies. 32 SNPs were included for further analysis. We selected 10 MI-associated SNPs. The average *F*-statistic value was 226.94. 1 MI-associated SNPs were not available in the summary data of fatigue and without proxy SNP. In the harmonization process, 1 SNP (rs4007642) was excluded for being palindromic. 8 MI-associated SNPs were included in the final analysis. 12 CHD-associated SNPs were included in the analysis with an average *F*-statistic value of 158.57. 1 SNP was not available in the fatigue GWAS data. 2 palindromic SNPs were excluded (rs4007642, rs4766578). More information for selected SNPs was displayed in Supplementary Tables S2–S4.

### 3.2. Causal effects of fatigue on CVD

**Figure 2:**
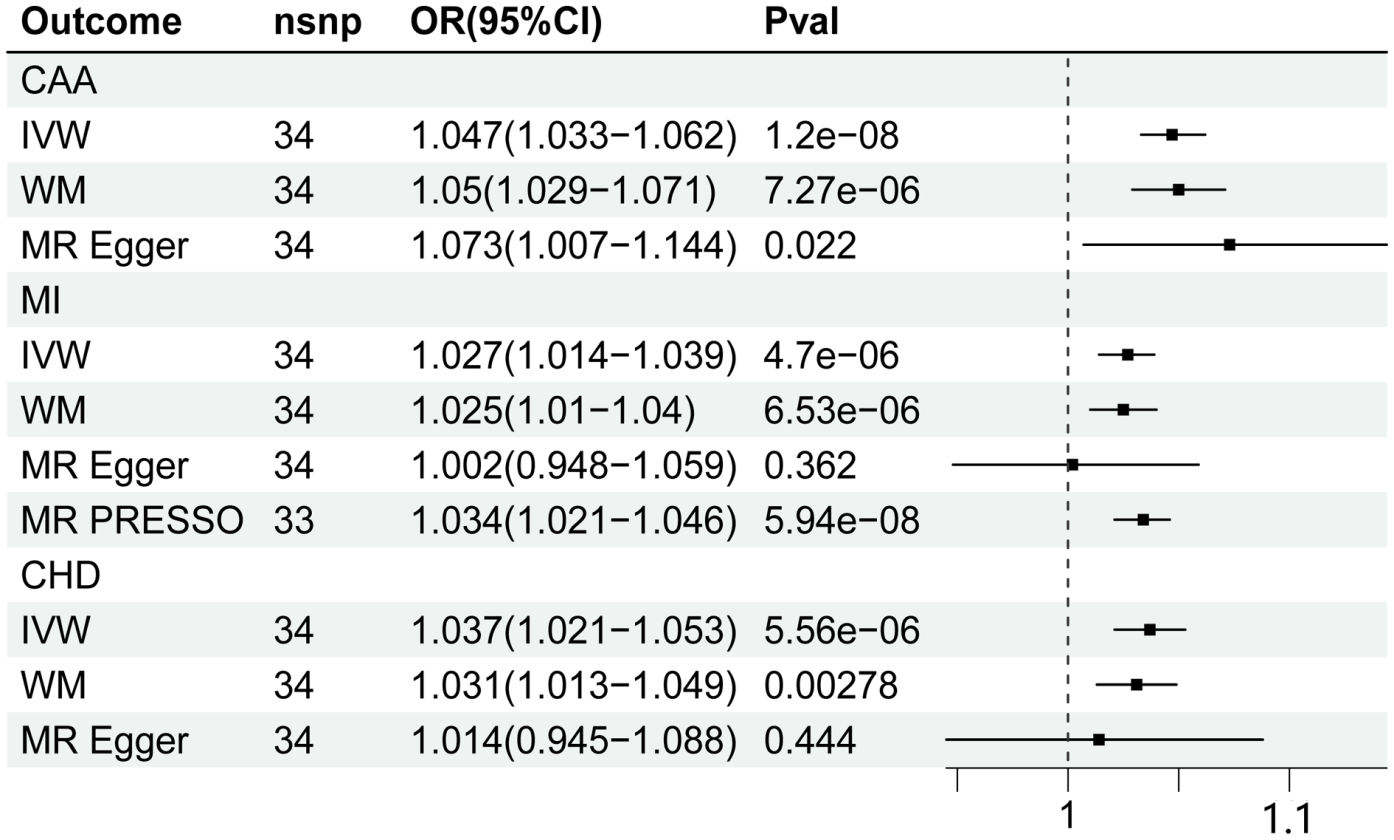
Forest plot of Mendelian randomization analyses for the relevance of fatigue with risk of CAA, CHD, and MI. CAA, coronary artery atherosclerosis; MI, myocardial infarction; CHD, coronary heart disease; nsnp, numbers of single-nucleotide polymorphism; IVW, inverse variance weighted method; OR, odds ratio; CI, confidential interval.

The MR estimates were shown in Figure 2. In our primary analysis, genetically predicted fatigue was associated with a higher risk of CAA (OR 1.047, 95%CI 1.033–1.062), which was consistent with the findings of MR Egger regression and weighted median. No evidence for heterogeneity and pleiotropy was shown (*p* > 0.05). Fatigue was also associated with an increased risk of MI (OR 1.027 95%CI 1.014–1.039). The weighted median result supports the conclusion, although the causal estimate from the MR-Egger regression was not significant. Analysis of heterogeneity and horizontal pleiotropy was displayed in Table 2. Cochran’s Q statistic showed heterogeneity in the result. Thus, we used IVW random effect model as the main analysis. MR-PRESSO test revealed potential horizontal pleiotropy (*p* < 0.05) and detected outliers (rs111657181). After the elimination of outliers, the corrected results are still significant (OR 1.034 95%CI 1.021–1.046). According to our MR analysis, fatigue could increase the risk of CHD (OR 1.037, 95%CI 1.021–1.053). The result of MR Egger was not consistent. Due to the substantial heterogeneity, we chose the IVW random-effect model. Although the MR-PRESSO result indicated horizontal pleiotropy (*p* < 0.05), no significant outliers were detected. The causal relationship between fatigue and CHD should be interpreted cautiously. The results of leave-one-out analyses did not indicate the effects were influenced by single SNPs. The leave-one-out analysis plots and the funnel plots were shown in Supplementary Figures S1, S2.

**Table 2:** Pleiotropy and heterogeneity test results of causal relationship between fatigue and coronary artery disease.

Exposures	Outcomes	MR-Egger		MR-PRESSO		Cochran’s Q (IVW)	
		Intercept	*p*-value	outlier	*p*-value	Q	*p*-value
Fatigue	CAA	−0.001	0.093	NA	0.192	41.498	0.147
	MI	−0.0003	0.697	1	0.011/0.114*	54.106	0.012
	CHD	−0.0002	0.805	NA	0.001	64.189	<0.001
CAA	Fatigue	−0.002	0.168	NA	0.002	55.881	0.004
MI		−0.001	0.621	NA	0.045	15.405	0.031
CHD		−0.001	0.529	NA	0.131	11.482	0.176

*: *p*-value for horizontal pleiotropy before and after removal of significant outliers. CAA, coronary artery atherosclerosis; MI, myocardial infarction; CHD, coronary heart disease; MR-PRESSO, Mendelian randomization pleiotropy residual sum and outliers; IVW, inverse variance weighted method.

### 3.3. Causal effects of CVD on fatigue

**Figure 3:**
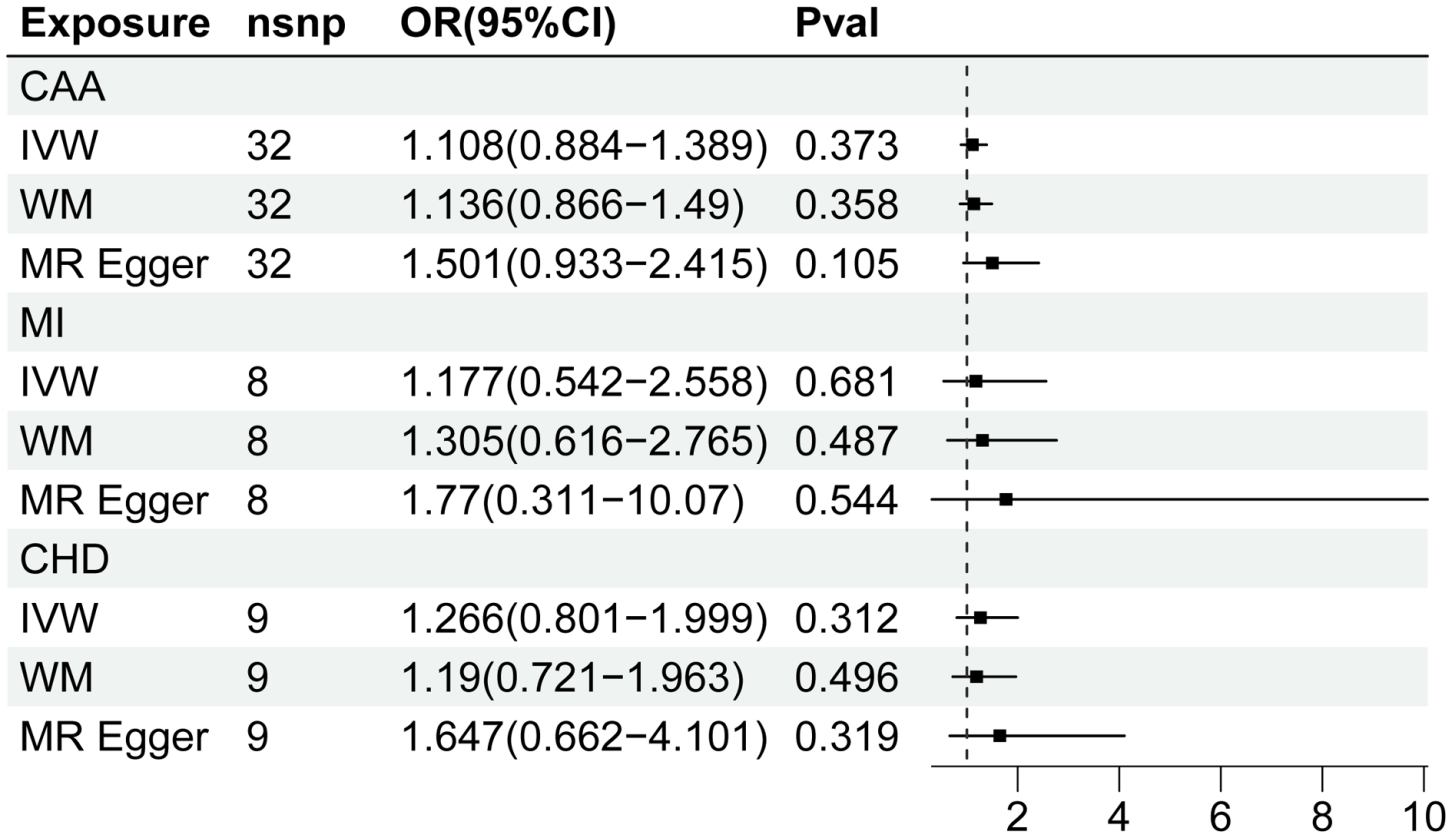
Forest plot of Mendelian randomization analyses for the relevance of coronary artery disease with risk of fatigue. CAA, coronary artery atherosclerosis; MI, myocardial infarction; CHD, coronary heart disease; nsnp, numbers of single-nucleotide polymorphism; IVW, inverse variance weighted method; OR, odds ratio; CI, confidential interval.

The MR estimate of different methods was presented in Figure 3. There was no causal association between genetically predicted CVD and fatigue. Cochran Q statistic showed heterogeneity for the causal estimate of CAA and MI with fatigue. Thus, IVW random-effect model was adopted. Additionally, the results of the causal estimate of CAA and MI on fatigue were suggested to have possible horizontal pleiotropy by the MR-PRESSO test. No significant outliers were detected. The results of leave-one-out analyses confirmed the consistency of the effects. The leave-one-out analysis plot and funnel plots were shown in Supplementary Figures S3, S4.

## 4. Discussion

The MR analysis explored the causal relationship between self-reported fatigue and CAD. The bidirectional analysis guaranteed the inference of causality in both directions. Our results indicated that genetically predicted fatigue was associated with a higher risk of CAD in individuals in Europe. The reverse MR analysis found no evidence that genetic liability to CAD was related to fatigue.

Conventionally, self-reported fatigue was considered a post-CAD symptom. The study indicated that higher post-CAD fatigue was associated with increased mortality (7). Few studies investigated the association between fatigue and cardiovascular health in the general population. An early cohort study focused on World War II veteran twins indicated that prolonged fatigue was strongly associated with both risk of MI and CHD after adjustment for traditional risk factors (22). A large-scale prospective cohort study further revealed that participants with higher fatigue were at greater risk of cardiovascular disease-related death (23). For specific diseases, highly fatigue patients under hemodialysis therapy were proved to exhibit a higher risk of cardiovascular events (24). Furthermore, analysis of the CORONA trial showed that the severity of fatigue was associated with an increased risk of cardiovascular death or hospital stay in participants with heart failure (25). Since the concept of fatigue partially overlapped with job burnout and vital exhaustion, clinical evidence about their associations with CAD could indirectly indicate the impact of fatigue (26, 27). However, most studies discussed the association between fatigue and the prognosis of cardiovascular disease. Besides, it is hard to avoid confounding factors and reverse causality in observational studies. Therefore, based on the summary level of GWAS data, this is the first study to confirm the causal relationship between self-reported fatigue and the risk of CAD.

Fatigue is a multidimensional phenomenon that involves both mental and physical components (13). Although little research concentrates on the mechanism linking self-reported fatigue to CAD, possible explanations for the association between mood disorder and cardiovascular disease could be referred. A previous review concluded three common mechanisms between mood disorder and cardiovascular dysregulation (28). Neuroendocrine alterations including activation of the hypothalamic–pituitary–adrenal (HPA) axis and renin-angiotensin-aldosterone system (RAAS) are believed to play an important role in the disease progression process. Besides, immune dysregulation and further activation of proinflammatory responses could promote the development of both diseases. The third mechanism is the imbalance of the autonomic nervous system. Several studies showed that fatigue is associated with a lower level of cardiovascular reactivity. Evidence from a cross-sectional study indicated that blood pressure responses to stress were inversely associated with fatigue level (29). A recent study further pointed out that cardiovascular reactivity to anticipatory stress was inversely associated with both global fatigue and mental fatigue, even after adjusting for covariates (30). The decline of cardiovascular reactivity is considered accompanied by poor state of health. Previous study showed that lower heart rate reactivity were associated with deteriorated physical disability (31). Nevertheless, the impact of decreased cardiovascular reactivity to CAD is unclear yet. The last possible mechanism is the disrupted central neurotransmitter systems. Disruptions of norepinephrine, dopamine, and serotonergic system may be the common pathophysiology of mood disorder and CVD, which required further studying. The relationship between fatigue and sleep should not be ignored. The latest study reported that both physical and psychological fatigue is associated with sleep quality (32). A systematic review concluded that there is a significant correlation between short sleep duration, fatigue, and increased incidence of CHD (33). Since most of the explanations lack supporting evidence, the mechanism between fatigue and CAD still deserves exploration.

The strengths of our study include two aspects. This is the first MR analysis to assess the causal relationship between self-reported fatigue and CAD. Moreover, we conducted a bidirectional MR analysis to avoid reverse causality and confirmed the findings of observational studies with less bias. However, our study has several limitations. Firstly, the MR-PRESSO test showed potential horizontal pleiotropy for several analysis data, but no outliers were detected. As a result, additional multivariable MR analysis should be carried out, and the findings should be evaluated cautiously. Secondly, we only used GWAS data from European ancestry, and the deficiency of GWAS from other populations limits the generality of our results. Thirdly, we did not discuss the causal relationship between other somatic depressive symptoms and CAD. Further studies could focus on this blank area. Fourthly, the mechanism of the association is yet unclear. Therefore, further fundamental studies should be completed.

Based on the above discussions, future studies could be implemented towards following aspects. To validate the causal association, a multivariable MR study adjusted for conventional risk factors for CAD should be conducted. Besides, GWAS data from different cohorts should be used to confirm the robustness and generality of the results. As fatigue is only one of the somatic depressive symptoms, the associations between other specific symptoms including appetite loss, sleep disorder, and CAD could be studied to improve the quality of research. To translate the result into clinical practice, randomized-control trials about the effect of intervention for fatigue and risk of CAD are required.

## 5. Conclusion

In conclusion, our MR study supported a causal relationship between self-reported fatigue and CAD. There was no evidence of reverse causality. The outcome was consistent with previous observational studies and provided more evidence about the involvement of fatigue in the development of CAD. The consistency of our findings needs to be confirmed by other GWAS studies.

## Data availability statement

The original contributions presented in the study are included in the article/Supplementary material, further inquiries can be directed to the corresponding author.

## Author contributions

XQ, SW, and QH contributed to the study design and data analysis. XQ, LQ, and XC drafted the manuscript giving contributed to the table, figures, and text editing. KO and YC revisited the article implementing the final manuscript form. All authors contributed to the article and approved the submitted version.

## Funding

This work was supported by Shenzhen Science and Technology Innovation Foundation (No. JCYJ20180228162359914) and Guangdong-Shenzhen Joint Fund Youth Project (No. 2021A1515111110).

## Conflict of interest

The authors declare that the research was conducted in the absence of any commercial or financial relationships that could be construed as a potential conflict of interest.

## Publisher’s note

All claims expressed in this article are solely those of the authors and do not necessarily represent those of their affiliated organizations, or those of the publisher, the editors and the reviewers. Any product that may be evaluated in this article, or claim that may be made by its manufacturer, is not guaranteed or endorsed by the publisher.
