# Supplementary material for: Causal association between self-reported fatigue and coronary artery disease: a bidirectional two-sample Mendelian randomization analysis
Source: Front Psychiatry. 2023 Sep 20;14:1166689. doi: 10.3389/fpsyt.2023.1166689 (PMC10547863; doi:10.3389/fpsyt.2023.1166689)
Supplement: Supplementary file 1 [file Data_Sheet_1.PDF]

```
#----fatigue on CAD-----
setwd("D:/underreview/fatigue")
library(TwoSampleMR)
library(MRPRESSO)
#---data0 is provided in Supplementary table 1(selected SNP for fatigue/ukb-b-929 with F-statistic>=10)------
exp_d <- read_exposure_data(
   filename = 'data0.csv',
   clump = FALSE,
   sep= ",",
   snp_col = "SNP",
   beta_col = "beta.exposure",
   se_col = "se.exposure",
   effect_allele_col ="effect_allele.exposure",
   other_allele_col = "other_allele.exposure",
   eaf_col = "eaf.exposure",
   pval_col = "pval.exposure"
)

IS_out_dat<-extract_outcome_data(
   snps = exp_d$SNP,
   outcomes = "ukb-d-I9_CHD/ukb-d-I9_CORATHER/ukb-d-I9_MI",
   proxies = T,
   rsq = 0.8,
   palindromes=1,
   maf_threshold = 0.3
)

dat<-harmonise_data(exposure_dat = exp_d,outcome_dat = IS_out_dat)

res<-mr(dat,method_list = c("mr_ivw","mr_weighted_median","mr_egger_regression"))

generate_odds_ratios(res)

mr_heterogeneity(dat)

mr_pleiotropy_test(dat)

res_loo<-mr_leaveoneout(dat)

res_single<-mr_singlesnp(dat)

p1<-mr_funnel_plot(res_single)
p1[[1]]
```

```
p2<-mr_leaveoneout_plot(res_loo)
p2[1]]

mr_presso(BetaOutcome ="beta.outcome", BetaExposure = "beta.exposure", SdOutcome ="se.outcome", SdExposure = "se.exposure",
          OUTLIERtest = TRUE,DISTORTIONtest = TRUE, data = dat, NbDistribution = 1000,
          SignifThreshold = 0.05)

#---CAD on fatigue----

exp_dat_clumped<-extract_instruments(outcomes = "ukb-d-I9_CHD/ukb-d-I9_CORATHER/ukb-d-I9_MI",
                                     p1=5e-8,access_token = NULL,clump = T,
                                     r2=0.01,kb=10000)

IS_out_dat<-extract_outcome_data(
  snps = exp_d$SNP,
  outcomes = "ukb-b-929",
  proxies = T,
  rsq = 0.8,
  palindromes=1,
  maf_threshold = 0.3
)

dat<-harmonise_data(exposure_dat = exp_dat_clumped,outcome_dat = IS_out_dat)

res<-mr(dat,method_list = c("mr_ivw","mr_weighted_median","mr_egger_regression"))

generate_odds_ratios(res)

mr_heterogeneity(dat)

mr_pleiotropy_test(dat)

res_loo<-mr_leaveoneout(dat)

res_single<-mr_singlesnp(dat)

p1<-mr_funnel_plot(res_single)
p1[[1]]

p2<-mr_leaveoneout_plot(res_loo)
p2[1]]
```

```
mr_presso(BetaOutcome ="beta.outcome", BetaExposure = "beta.exposure", SdOutcome
="se.outcome", SdExposure = "se.exposure",
          OUTLIERtest = TRUE,DISTORTIONtest = TRUE, data = dat, NbDistribution = 1000,
          SignifThreshold = 0.05)
```
