# Supplementary material for: Causal association between self-reported fatigue and coronary artery disease: a bidirectional two-sample Mendelian randomization analysis
Source: Front Psychiatry. 2023 Sep 20;14:1166689. doi: 10.3389/fpsyt.2023.1166689 (PMC10547863; doi:10.3389/fpsyt.2023.1166689)

Causal association between self-reported fatigue and coronary artery disease: a bidirectional two-sample mendelian randomization analysis

# Supplementary Tables

**Supplementary Table 1.** Information of instrumental variables utilized in the Mendelian Randomization for fatigue on coronary artery disease

| chr | pva | se | beta | pos | SNP | A1 | A2 | eaf | F |
| --- | --- | --- | --- | --- | --- | --- | --- | --- | --- |
| 1 | 1.90E-10 | 0.001834 | 0.011678 | 2.02E+08 | rs2820309 | G | A | 0.344964 | 15.57742 |
| 1 | 4.40E-09 | 0.001752 | 0.010284 | 1.15E+08 | rs10732635 | G | T | 0.445322 | 16.30221 |
| 1 | 4.40E-08 | 0.001882 | -0.0103 | 96685378 | rs17374635 | C | T | 0.31835 | 15.1798 |
| 1 | 1.00E-08 | 0.001749 | -0.01001 | 1.74E+08 | rs6425280 | G | A | 0.462613 | 16.3325 |
| 2 | 3.50E-08 | 0.002442 | 0.013463 | 24112559 | rs111657181* | C | G | 0.150183 | 11.70113 |
| 3 | 1.40E-08 | 0.001991 | 0.011303 | 18674644 | rs4619804 | C | A | 0.737279 | 14.35202 |
| 3 | 2.80E-08 | 0.001749 | 0.009711 | 1.73E+08 | rs529200 | G | A | 0.527591 | 16.33829 |
| 3 | 5.10E-12 | 0.002103 | -0.01451 | 49213637 | rs9586 | T | C | 0.778534 | 13.58662 |
| 3 | 1.00E-08 | 0.001747 | -0.01001 | 1.36E+08 | rs589092 | G | A | 0.506889 | 16.35663 |
| 3 | 2.70E-10 | 0.001754 | -0.01108 | 85371400 | rs818215 | C | A | 0.459631 | 16.28428 |
| 4 | 1.10E-08 | 0.001912 | -0.01092 | 66344954 | rs11936348 | T | C | 0.296337 | 14.94514 |
| 4 | 4.70E-09 | 0.002071 | -0.01213 | 25408838 | rs34811474 | A | G | 0.2307 | 13.79485 |
| 4 | 1.50E-10 | 0.001878 | -0.01204 | 3225371 | rs82334 | C | A | 0.315286 | 15.21124 |
| 5 | 2.30E-08 | 0.001763 | -0.00985 | 8115393 | rs4701803 | T | C | 0.56534 | 16.20889 |
| 5 | 1.00E-09 | 0.001852 | -0.0113 | 1.53E+08 | rs11744083 | C | A | 0.33171 | 15.4301 |
| 5 | 2.30E-10 | 0.00177 | -0.01121 | 1.52E+08 | rs34299746 | T | G | 0.421202 | 16.14413 |
| 5 | 8.80E-09 | 0.001861 | -0.01071 | 1.04E+08 | rs6421926 | C | T | 0.674978 | 15.35257 |
| 7 | 2.90E-08 | 0.001744 | 0.009673 | 1.09E+08 | rs62471111 | C | T | 0.477803 | 16.37876 |
| 7 | 2.10E-08 | 0.002412 | -0.01351 | 2083267 | rs62444907 | T | C | 0.155217 | 11.84557 |
| 8 | 9.20E-09 | 0.001746 | 0.01003 | 73890425 | rs4588900 | A | G | 0.51551 | 16.36187 |
| 9 | 2.80E-08 | 0.001836 | -0.01019 | 96351217 | rs10761238 | T | C | 0.351783 | 15.55892 |
| 10 | 8.70E-11 | 0.001801 | -0.01168 | 12685653 | rs12218281 | C | T | 0.622505 | 15.86337 |
| 11 | 4.60E-11 | 0.001784 | -0.01175 | 1.13E+08 | rs7131681 | T | C | 0.60186 | 16.01041 |
| 11 | 2.40E-08 | 0.001749 | 0.009767 | 89019737 | rs598769 | T | C | 0.469887 | 16.33213 |
| 11 | 8.40E-10 | 0.001857 | -0.01139 | 57498232 | rs2847308 | T | C | 0.671466 | 15.38747 |
| 12 | 5.50E-14 | 0.001846 | 0.013882 | 1.1E+08 | rs7316486 | G | A | 0.344116 | 15.47691 |
| 14 | 8.80E-10 | 0.002027 | -0.01242 | 47161757 | rs11625230 | C | T | 0.247512 | 14.09284 |
| 14 | 5.30E-12 | 0.001827 | 0.012598 | 69711200 | rs1464307 | A | G | 0.646713 | 15.6395 |
| 17 | 6.00E-09 | 0.001795 | 0.01044 | 50316131 | rs12453010 | T | C | 0.393187 | 15.91995 |
| 17 | 4.80E-13 | 0.002181 | -0.01577 | 2477787 | rs10438710 | G | A | 0.794406 | 13.10132 |
| 18 | 2.20E-09 | 0.001862 | -0.01115 | 35138245 | rs12967855 | G | A | 0.668905 | 15.33963 |
| 18 | 3.50E-11 | 0.00177 | 0.011726 | 50957922 | rs8089865 | A | G | 0.578514 | 16.13674 |
| 18 | 1.30E-09 | 0.001864 | 0.011308 | 52765283 | rs56403421 | C | A | 0.331583 | 15.3272 |
| 22 | 3.50E-09 | 0.001868 | 0.011026 | 41713111 | rs9611555 | A | G | 0.324784 | 15.29749 |

SNP, single nucleotide polymorphism; A1, effect allele; A2, other allele; se, standard error; * significant outliers detected by MR-PRESSO test

**Supplementary Table 2.** Information of instrumental variables utilized in the Mendelian Randomization for coronary artery atherosclerosis on fatigue

| chr | pval | se | beta | pos | SNP | A1 | A2 | F |
| --- | --- | --- | --- | --- | --- | --- | --- | --- |
| 2 | 4.44E-08 | 0.00072 | 0.00394 | 1.65E+08 | rs10172929 | G | T | 39.52872 |
| 4 | 7.74E-11 | 0.000651 | 0.004233 | 1.48E+08 | rs10305838 | C | T | 43.71641 |
| 12 | 7.30E-09 | 0.000456 | -0.00264 | 1.12E+08 | rs10774624 | A | G | 62.31368 |
| 1 | 1.34E-09 | 0.00171 | -0.01037 | 55505647 | rs11591147 | T | G | 16.67552 |
| 13 | 1.57E-10 | 0.000722 | -0.00462 | 1.11E+08 | rs11617955 | A | T | 39.41713 |
| 15 | 1.02E-12 | 0.000526 | -0.00375 | 79054108 | rs57708073* | G | A | 53.9853 |
| 16 | 2.16E-08 | 0.00046 | 0.002577 | 75482860 | rs12927562 | A | G | 61.68635 |
| 9 | 1.01E-73 | 0.000451 | 0.008196 | 22103813 | rs1333042 | G | A | 62.9356 |
| 19 | 1.95E-08 | 0.001339 | 0.007519 | 33959431 | rs145436496 | A | G | 21.293 |
| 2 | 4.08E-10 | 0.000683 | 0.004271 | 2.04E+08 | rs72934545* | T | G | 41.64504 |
| 3 | 2.86E-08 | 0.000626 | 0.003477 | 1.54E+08 | rs1620712 | G | C | 45.39858 |
| 2 | 9.31E-09 | 0.000902 | 0.00518 | 19942473 | rs16986953 | A | G | 31.57369 |
| 6 | 2.28E-34 | 0.001871 | 0.022875 | 1.61E+08 | rs186696265 | T | C | 15.24601 |
| 2 | 4.93E-12 | 0.000454 | 0.003133 | 85767735 | rs2028900 | T | C | 62.60662 |
| 7 | 1.22E-10 | 0.00063 | 0.004056 | 19049388 | rs2107595 | A | G | 45.13696 |
| 1 | 2.80E-10 | 0.0005 | 0.003154 | 2.23E+08 | rs2133189 | T | C | 56.82335 |
| 11 | 8.26E-11 | 0.000504 | -0.00327 | 1.04E+08 | rs2839812 | A | T | 56.40539 |
| 21 | 2.76E-12 | 0.000681 | 0.00476 | 35593827 | rs28451064 | A | G | 41.77781 |
| 8 | 2.32E-10 | 0.000463 | -0.00293 | 1.27E+08 | rs28601761 | G | C | 61.36764 |
| 12 | 4.92E-10 | 0.000882 | -0.00549 | 95547732 | rs10859843* | C | T | 32.28919 |
| 6 | 4.59E-08 | 0.000509 | -0.00278 | 1.61E+08 | rs3861973 | T | C | 55.82208 |
| 10 | 4.21E-09 | 0.000457 | 0.002684 | 1.24E+08 | rs760337* | A | G | 62.15478 |
| 6 | 5.64E-70 | 0.000826 | 0.014607 | 1.61E+08 | rs55730499 | T | C | 34.47393 |
| 1 | 3.43E-23 | 0.000546 | 0.005417 | 1.1E+08 | rs602633 | G | T | 52.04974 |
| 1 | 1.81E-09 | 0.000781 | -0.0047 | 56963627 | rs72664318 | G | A | 36.42499 |
| 16 | 2.30E-11 | 0.000523 | -0.00349 | 83045790 | rs7500448 | G | A | 54.3641 |
| 19 | 1.55E-11 | 0.000677 | 0.004566 | 45410002 | rs769449 | A | G | 42.01555 |
| 19 | 7.38E-11 | 0.000721 | -0.0047 | 11190481 | rs61194703* | T | A | 39.47334 |
| 15 | 5.10E-13 | 0.000453 | 0.003273 | 91422543 | rs8039305 | C | T | 62.64974 |
| 19 | 6.23E-09 | 0.000512 | -0.00298 | 41822986 | rs8109627 | C | T | 55.49016 |
| 6 | 5.46E-10 | 0.000495 | -0.00307 | 1.34E+08 | rs9285476 | G | C | 57.34407 |
| 6 | 3.65E-19 | 0.000459 | 0.004109 | 12903957 | rs9349379 | G | A | 61.8264 |

SNP, single nucleotide polymorphism; A1, effect allele; A2, other allele; se, standard error; *rs57708073, rs72934545, rs10859843, rs760337, rs61194703 were used as proxy SNP (linkage disequilibrium R2>0.8) in the summary statistics of the outcome.

**Supplementary Table 3.** Information of instrumental variables utilized in the Mendelian Randomization for fatigue on myocardial infarction

| chr | pval | se | beta | pos | SNP | A1 | A2 | F |
| --- | --- | --- | --- | --- | --- | --- | --- | --- |
| 6 | 3.12E-28 | 0.000592 | 0.006522 | 1.61E+08 | rs10455872 | G | A | 168.9529 |
| 12 | 6.29E-13 | 0.000322 | -0.00232 | 1.12E+08 | rs10774625 | G | A | 310.2056 |
| 1 | 5.54E-09 | 0.000388 | -0.00226 | 1.1E+08 | rs12740374 | T | G | 257.8364 |
| 2 | 9.02E-10 | 0.000488 | 0.002991 | 2.04E+08 | rs72934545* | T | G | 204.7775 |
| 6 | 1.91E-16 | 0.001337 | 0.011004 | 1.61E+08 | rs186696265 | T | C | 74.76743 |
| 1 | 4.34E-08 | 0.000346 | 0.001895 | 2.23E+08 | rs1909196 | C | T | 288.981 |
| 21 | 9.09E-09 | 0.000487 | 0.002798 | 35593827 | rs28451064 | A | G | 205.4292 |
| 6 | 6.40E-10 | 0.000328 | 0.002029 | 12903957 | rs9349379 | G | A | 304.5957 |

SNP, single nucleotide polymorphism; A1, effect allele; A2, other allele; se, standard error;

* rs72934545 were used as proxy SNP (linkage disequilibrium R2>0.8) in the summary statistics of the outcome.

**Supplementary Table 4.** Information of instrumental variables utilized in the Mendelian Randomization for fatigue on coronary heart disease

| chr | pval | se | beta | pos | SNP | A1 | A2 | F |
| --- | --- | --- | --- | --- | --- | --- | --- | --- |
| 6 | 4.08E-41 | 0.000707 | 0.009497 | 1.61E+08 | rs10455872 | G | A | 117.3866 |
| 1 | 1.18E-12 | 0.000463 | -0.00329 | 1.1E+08 | rs12740374 | T | G | 178.7644 |
| 17 | 1.95E-08 | 0.00043 | 0.002413 | 66391826 | rs12939053 | A | C | 192.7315 |
| 2 | 6.24E-09 | 0.000583 | 0.00339 | 2.04E+08 | rs72934545* | T | G | 142.1473 |
| 6 | 3.94E-22 | 0.001598 | 0.015457 | 1.61E+08 | rs186696265 | T | C | 52.0583 |
| 21 | 8.98E-10 | 0.000582 | 0.003563 | 35593827 | rs28451064 | A | G | 142.5991 |
| 8 | 3.37E-10 | 0.000442 | -0.00277 | 19870263 | rs35617716 | A | T | 187.473 |
| 15 | 1.41E-09 | 0.000408 | 0.002468 | 91404705 | rs4932370 | A | G | 203.0317 |
| 6 | 1.05E-10 | 0.000392 | 0.002534 | 12903957 | rs9349379 | G | A | 210.9523 |

SNP, single nucleotide polymorphism; A1, effect allele; A2, other allele; se, standard error; *rs72934545 were used as proxy SNP (linkage disequilibrium R2>0.8) in the summary statistics of the outcome.

# Supplementary Figures

**Figure S1**. Funnel plot of the causal relationships between fatigue and coronary artery disease. (A) MR estimate for fatigue on coronary artery atherosclerosis. (B) MR estimate for on myocardial infarction. (C) MR estimate for on coronary heart disease. The funnel plot illustrated the overall symmetry of causal estimates across all instrumental variables.


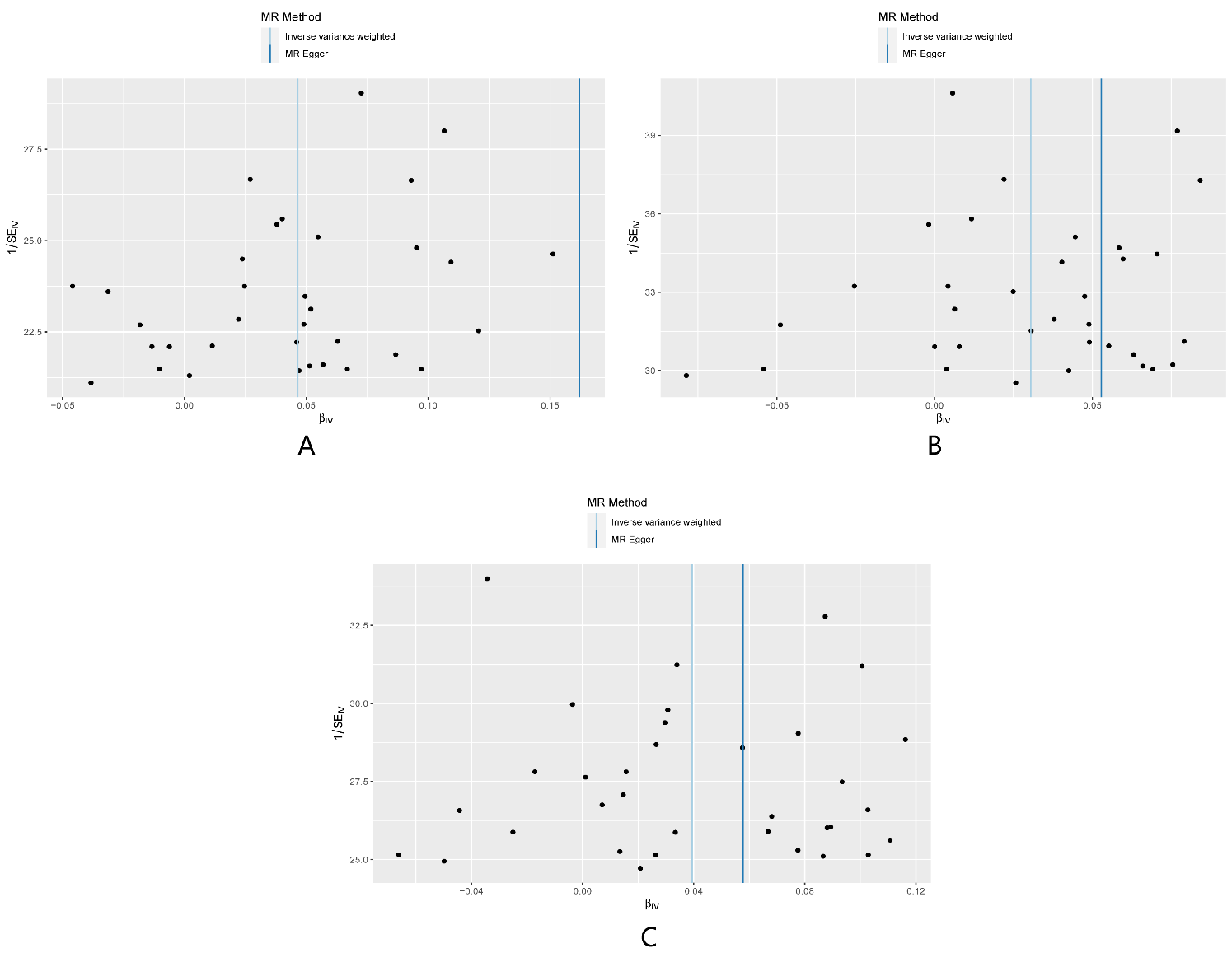


**Figure S2.** Leave-one-out plot of the causal relationships between fatigue and coronary artery disease. (A) MR estimate for fatigue on coronary artery atherosclerosis. (B) MR estimate for on myocardial infarction. (C) MR estimate for on coronary heart disease. The leave-one-out plot visualized how the causal estimates (point with horizontal line) between fatigue and coronary artery disease were not influenced by the removal of single variant.


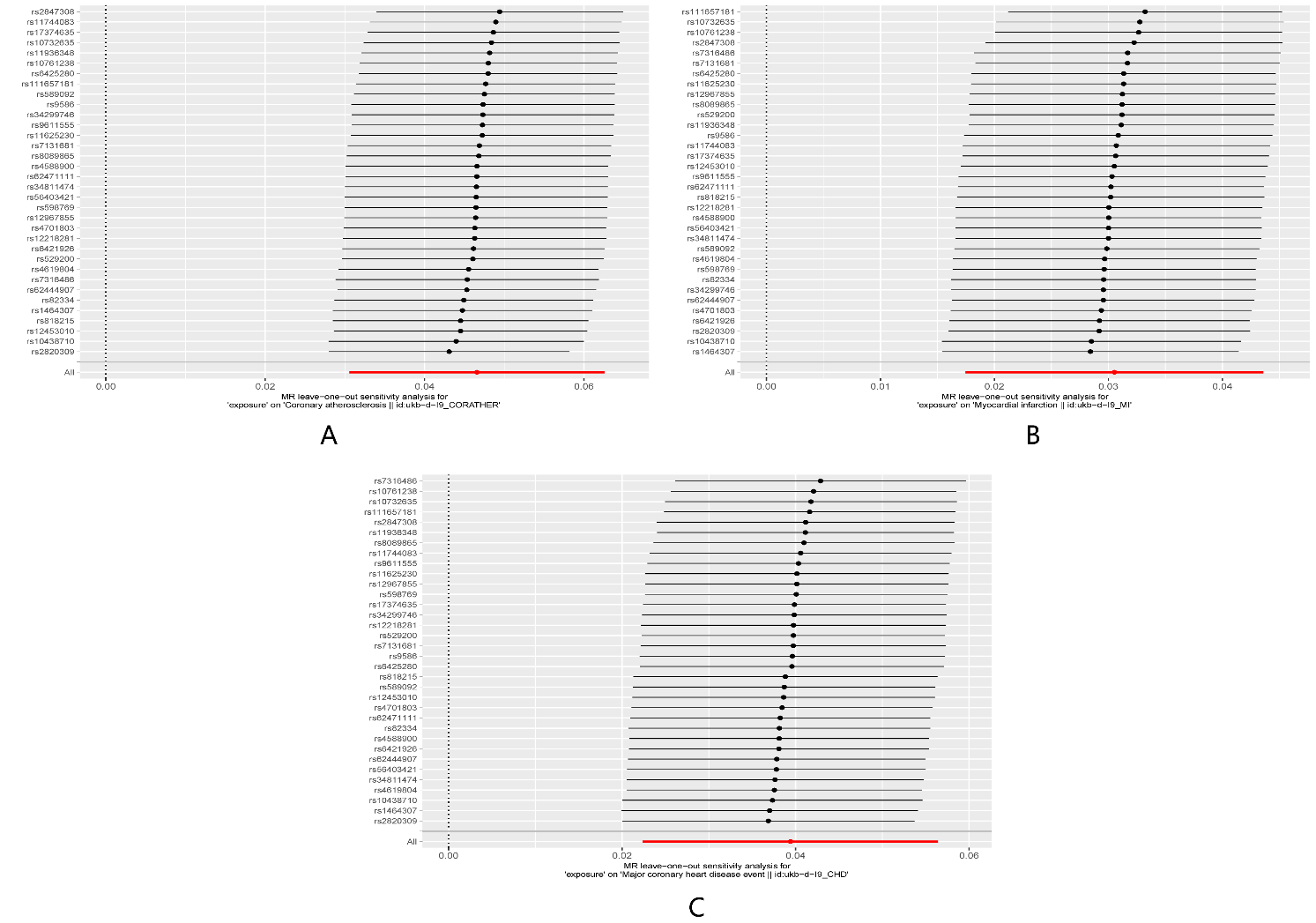


**Figure S3**. Funnel plot of the causal relationships between fatigue and coronary artery disease. (A) MR estimate for coronary artery atherosclerosis on fatigue. (B) MR estimate for myocardial infarction on fatigue. (C) MR estimate for coronary heart disease on fatigue. The funnel plot illustrated the overall symmetry of causal estimates across all instrumental variables.


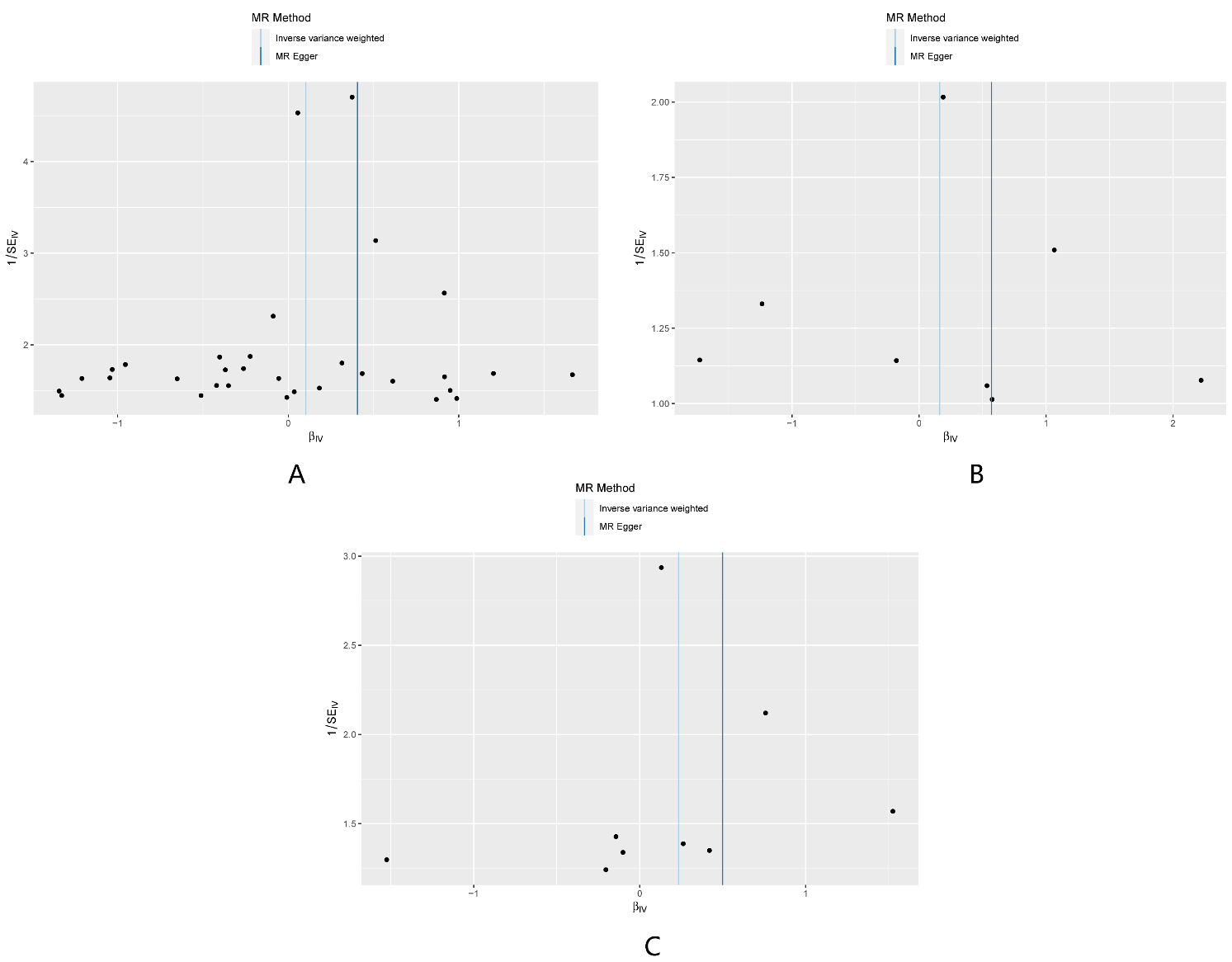


**Figure S4**. Leave-one-out plot of the causal relationships between fatigue and coronary artery disease. (A) MR estimate for coronary artery atherosclerosis on fatigue. (B) MR estimate for coronary heart disease on fatigue. (C) MR estimate for myocardial infarction on fatigue. The leave-one-out plot visualized how the causal estimates (point with horizontal line) between fatigue and coronary artery disease were not influenced by the removal of single variant.


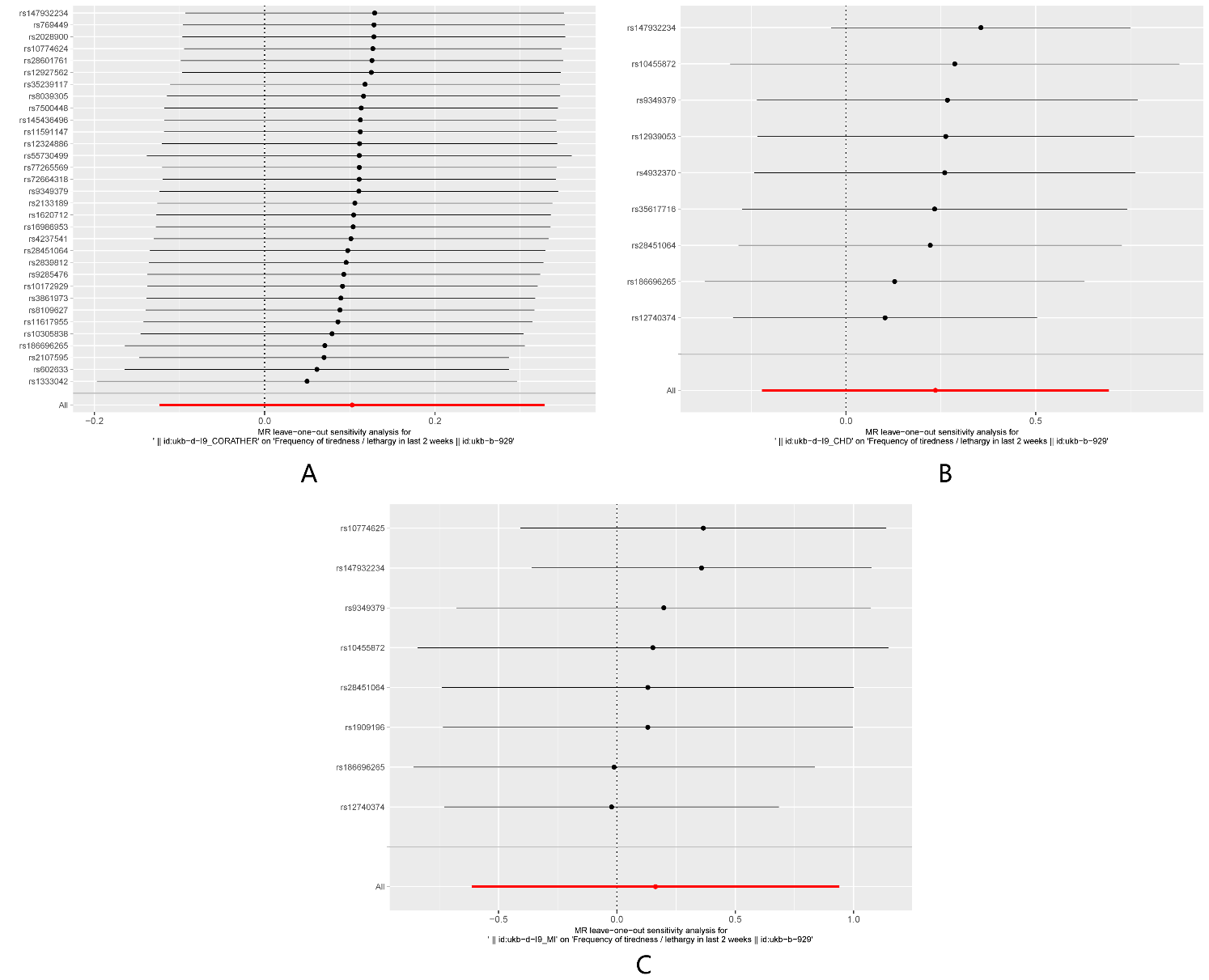

Supplement: Supplementary file 2 [file Data_Sheet_2.docx]
